# Thyroid hormones and the potential for regulating glucose metabolism in cardiomyocytes during insulin resistance and T2DM

**DOI:** 10.14814/phy2.14858

**Published:** 2021-08-17

**Authors:** Dora A. Mendez, Rudy M. Ortiz

**Affiliations:** ^1^ Department of Molecular & Cell Biology School of Natural Sciences University of California Merced CA USA

**Keywords:** fatty acid oxidation, glucose oxidation, GLUT4, mitochondrial biogenesis, T3

## Abstract

In order for the heart to maintain its continuous mechanical work and provide the systolic movement to uphold coronary blood flow, substantial synthesis of adenosine triphosphate (ATP) is required. Under normal conditions cardiac tissue utilizes roughly 70% fatty acids (FA), and 30% glucose for the production of ATP; however, during impaired metabolic conditions like insulin resistance and diabetes glucose metabolism is dysregulated and FA account for 99% of energy production. One of the major consequences of a shift in FA metabolism in cardiac tissue is an increase in reactive oxygen species (ROS) and lipotoxicity, which ultimately lead to mitochondrial dysfunction. Thyroid hormones (TH) have direct effects on cardiac function and glucose metabolism during impaired metabolic conditions suggesting that TH may improve glucose metabolism in an insulin resistant condition. None‐classical TH signaling in the heart has shown to phosphorylate protein kinase B (Akt) and increase activity of phosphoinositide‐3‐kinase (PI3K), which are critical mediators in the insulin‐stimulated glucose uptake pathway. Studies on peripheral tissues such as skeletal muscle and adipocytes have demonstrated TH treatment improved glucose intolerance in a diabetic model and increased insulin‐regulated glucose transporter (GLUT4) mRNA levels. GLUT4 is a downstream target of thyroid response element (TRE), which demonstrates that THs regulate glucose via GLUT4. Elevated 3,5,3′‐triiodothyronine (T3) increased glucose oxidation rate and decreased the glycolytic intermediate, fructose 6‐phosphate (F6P) in cardiomyocytes, in addition to increasing mitochondrial biogenesis and pyruvate transport across the mitochondrial membrane. These findings along with a few other studies on T3 treatment in cardiac tissue suggest TH may improve glucose metabolism in an insulin resistant model and ameliorate the effects of diabetes and metabolic syndrome. This review highlights the potential benefits of exogenous TH on ameliorating metabolic dysfunction in the heart.

## Graphical abstract

Under normal conditions cardiac tissue utilizes roughly 70% fatty acids (FA), and 30% glucose to produce ATP; however, during impaired metabolic conditions like insulin resistance and diabetes, glucose metabolism is dysregulated and FA can account for as much as 99% of energy production. Some of the major consequences of this shift toward greater FA metabolism include lipotoxicity, oxidative injury, inflammation and mitochondrial damage that can ultimately lead to cardiac remodeling. Thyroid hormones have been shown to increase GLUT4 mRNA expression in addition to colocalizing and increasing PI3K activity, which can potentially improve glucose metabolism under insulin resistant conditions.

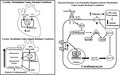

## 1 CARDIAC METABOLISM IN THE NORMAL HEART

In order for the heart to maintain its continuous mechanical work and provide the systolic movement to uphold coronary blood flow, substantial synthesis of ATP is required. The breakdown of ATP by myosin ATPase fuels the contractile shortening and regulates the ionic gradient across the sarcoplasmic reticulum to generate diastolic relaxation. The synthesis of ATP in cardiac tissue is regulated by oxidative phosphorylation in the mitochondria (Stanley, 2001). Under normal conditions, 70% to 90% of ATP generated in the heart comes from oxidation of FA while the remaining 10% to 30% is generated from glucose and, to a lesser extent, lactate oxidation under fasted conditions (Doenst et al., 2013). However, in fed conditions, there's an increase in plasma glucose oxidation.

### 1.1 FA and glucose metabolism

**FIGURE 1:**
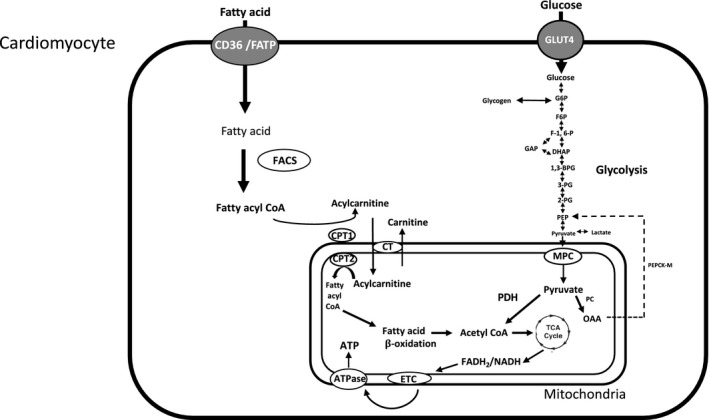
Schematic diagram of FA and glucose metabolism in the heart. FA enter cardiomyocytes either through diffusion or transport proteins such as FA translocase (CD36) and FA transport protein (FATP). CoA synthetase (FACS) converts FA into fatty acyl CoA. Fatty acyl CoA can either be esterified into lipid intermediates such as TAG or be converted to acylcarnitine via carnitine palmitoyl transferase 1 (CPT1). A majority of the fatty acyl CoA enters the β‐oxidation pathway which eventually leads to the production of ATP. Glucose enters the cardiomyocytes through GLUT‐family transporters. Glucose is phosphorylated by hexokinase II to produce glucose‐6‐phosphate (G6P), fructose 6‐phosphate (F6P), fructose 1, 6‐bisphosphate (F‐1,6‐P), glyceraldehyde 3‐phsophate (GAP), dihydroxyacetone phosphate (DHAP), 1,3‐bisphosphoglycerate (1,3‐BPG), 3‐phosphoglycerate (3‐PG), 2‐phosphoglycerate (2‐PG), phosphoenolpyruvate (PEP). Pyruvate then enters the mitochondria via mitochondrial pyruvate carrier (MPC), where it is converted to acetyl CoA by pyruvate dehydrogenase (PDHK), (Depre et al., 1999) or converted to oxaloacetate (OAA) by mitochondrial pyruvate carboxylase (PC). During starvation or diabetic conditions OAA can be converted back to phosphoenolpyruvate (PEP) by mitochondrial phosphoenolpyruvate carboxykinase (PEPCK‐M). Acetyl CoA then enters the TCA cycle generating reducing equivalents and ATP (Amaral & Okonko, 2015; Depre et al., 1999; Jitrapakdee et al., 2008; Fillmore & Lopaschuk, 2013; Aerni‐Flessner et al., 2012; Longo et al., 2006; Lopaschuk et al., 2010; Berg et al., 2002).

Both FA and glucose are used by the mitochondria to produce ATP via tricarboxylic acid (TCA) cycle, which generates the reducing equivalents: NADH and FADH_2_. These reducing equivalents release electrons to the electron transport chain (ETC) to produce ATP (Figure 1). The heart can switch between substrates depending on their availability and the energetic status of the cell. An increase in FA utilization suppresses glucose utilization, and an increase in glucose utilization can suppress FA uptake; this reciprocal inhibition is known as the 'Randle Cycle' (Amaral & Okonko, 2015). Impairment at any one of these enzymatic steps can reduce efficiency and impair mitochondrial function, decreasing β‐oxidation and contributing to the development of diabetic cardiomyopathies.

## 2 SHIFTS IN CARDIAC METABOLISM DURING INSULIN RESISTANCE

### 2.1 Cardiac metabolism is susceptible to diabetic conditions

Studies reveal that mortality rates among diabetic patients remain higher than those of the general population, with cardiovascular disease (CVD) being the most prevalent cause (Leon & Maddox, 2015). As previously stated, under ordinary conditions cardiac tissue utilizes roughly 70% FA and 30% glucose for the production of ATP; however, in a diabetic condition glucose metabolism is compromised and FA account for roughly 99% of energy production (Gamble & Lopaschuk, 1994; Randle, 1986). During insulin resistance reduced insulin action increases plasma FA and triglycerides (TG) storage (Schummer et al., 2008). This elevation in FA activates peroxisome proliferator activated receptor‐alpha (PPAR‐α) and amplifies FA β‐oxidation, attenuating glucose metabolism. Excess FA also inhibits glucokinase, phosphofructokinase 1 (PFK‐1), and pyruvate dehydrogenase kinase (PDHK) (Schummer et al., 2008). These studies reveal elevated plasma FA, TG, and myocyte lipid accumulation lead to the development of diabetic cardiomyopathy.

### 2.2 Elevated FA levels increases oxidative stress

One of the major consequences of a shift in FA metabolism in cardiac tissue is an increase of ROS and lipotoxicity, which ultimately lead to mitochondrial dysfunction. Accumulation of lipid, TG storage, and FA overload induces lipotoxicity. Elevated FA increased the expression lipoprotein lipase (LPL) and FA transport protein (FATP) leading to an increase in FA utilization and eventually diabetic cardiomyopathy (An & Rodrigues, 2006). ROS are generated in the mitochondria as a by‐product of aerobic respiration. When the production of ROS is greater than its removal, the heart undergoes oxidative stress, which leads to myocyte apoptosis and heart failure (Hafstad et al., 2013). Therefore, this maladaptation of a pronounced shift in FA utilization for cardiac metabolism contributes to the development of diabetic cardiomyopathies and heart failure. Recent findings have demonstrated that TH may ameliorate these effects during diabetic cardiomyopathy.

## 3 SEARCH METHODOLOGY

PubMed, Google Scholar, the California Digital Library, and the American Journal of Physiology (AJP) were searched using a combination of the following terms: “thyroid hormone,” “T4,” “T3,” “diabetic cardiomyopathy,” “insulin resistance,” “type 2 diabetes,” “metabolic syndrome,” “metabolism,” “glucose metabolism,” “glucose oxidation,” “glycolysis,” “glucose uptake,” “GLUT4,” and “insulin signaling.” Primary and review articles were the main sources used for this review. This review focuses on the potential of TH to regulate glucose metabolism during insulin resistance and type 2 diabetes (T2DM). Because the etiology of T2DM differs substantially from that of T1DM, studies of T1DM were not included here. Furthermore, studies and reviews on TH actions and glucose metabolism in T1DM already exist (Yuan et al., 2021; Liu & Severson, 1994; Biondi et al., 2019; Kosova et al., 2013), which include studies using streptozotocin (STZ)‐induced diabetic rodents, a model of T1DM, therefore, these studies were excluded from this review.

## 4 TH FUNCTION IN CARDIAC MUSCLE

**FIGURE 2:**
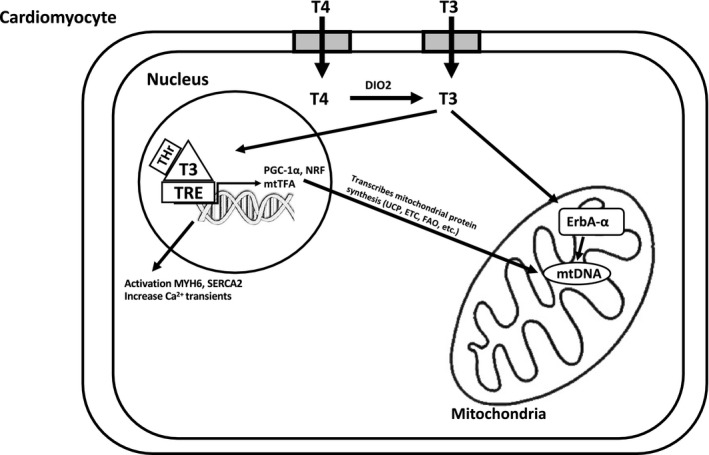
Schematic diagram of T3 genomic actions in cardiomyocyte mitochondrial biogenesis. TH binds to its receptor (TR) in the nucleus and forms a heterodimer retinoid X receptor (RXR) ‐TR complex. Once the ligand‐receptor complex binds to regulatory genes in the nucleus it activates the expression of myosin 6 (MYH6), which enhances contractility in the myofilaments. TH also activates sarcoplasmic/endoplasmic reticulum calcium ATPase 2 (SERCA2), which enhances Ca^2+^ transients that assist in systolic contraction and diastolic relaxation. In the nuclear compartment RXR‐TR complex initiates a large assortment of genes such as peroxisome proliferator‐activated receptor γ coactivator‐1α (PGC‐1α) and nuclear respiratory factors (NRF) all which modulate mitochondrial function and FA β‐oxidation. TH can also activate mitochondria transcription of mtDNA either through mitochondrial transcription factor A (mtTFA) or directly through its orphan receptor (ErbA‐α) found in the mitochondria (Janssen et al., 2017; Marín‐García, 2010, 2013; Enríquez et al., 1999)

Contraction and systolic function of the myocardium is regulated by TH activity. TH promote mitochondrial biogenesis and mitochondrial protein synthesis by binding to specific TH receptors found in the nuclear and mitochondrial compartments (Figure 2). Based on these findings T3 increases substrate metabolism in cardiac tissue to support contraction and systole, which is significant because cardiac contractility is energetically expensive. The heart's ability to shift between FA and glucose metabolism supports efficient cardiac pumping and contractility. Glucose metabolism is more energetically efficient than FAs, providing 54% greater energy production for each mole of O_2_ consumed (Kessler & Friedman, 1998). Therefore, alterations associated with TH status could impact cellular metabolism and energetics that can affect CV function.

## 5 TH AND THE INSULIN SIGNALING PATHWAY

Though the classical genomic actions of TH signaling via nuclear thyroid hormone receptor are well defined, little is known about their non‐classical, non‐genomic signaling pathways. It is clear now that non‐classical TH actions induce a variety of biological responses (Senese et al., 2014). In Sprague Dawley rats treated with levothyroxine (LT4), the phosphorylation of protein kinase B (p‐Akt) increased at serine 473 and threonine 308 residues in myocytes, both of which activate Akt (Kuzman et al., 2005). Similarly, treatment with T3 increased phosphorylation of Akt at both serine and threonine residues in pancreatic β cells and vascular smooth muscle cells (Carrillo‐Sepúlveda et al., 2010; Verga Falzacappa et al., 2007). A possible mechanism proposed by which TH can activate Akt is through its receptor, TRβ1, which has been found to exist in the cytoplasm and to colocalize with PI3K p85α suggesting that TRβ1 may mediate non‐genomic T3 actions (Verga Falzacappa et al., 2007). In addition, T3 increased PI3K activity also likely in TRβ1‐associated manner. TH may also stimulate hypoxia‐inducible factor (HIF‐1α) via the PI3 K/ERK pathway whose target genes include glucose transporter 1 (GLUT1), phosphofructokinase (PFK), and monocarboxylate transporter 4 (MCT4) all which regulate glucose metabolism and glucose uptake (Moeller et al., 2005). It is important to note that these studies were not under insulin resistant nor T2DM conditions, and thus, more detailed studies of cellular TH events during these conditions are warranted. Collectively, these findings suggest that the non‐genomic actions of TH on critical mediators of insulin‐stimulated glucose uptake such as PI3 K and Akt may complement the known classical TH actions or potentially compensate for the loss of those classical functions during conditions associated with metabolic derangements.

## 6 TH AND GLUCOSE METABOLISM

### 6.1 Recent literature on peripheral tissues

#### 6.1.1 Liver

The association between hyperthyroidism and increased plasma glucose has been well established. In a pharmacologic study, acute treatment with T3 (100 μg/kg) increased serum glucose and decreased hepatic glycogen levels in euthyroid Zucker fatty rats suggesting that T3 administration induced an increase in plasma glucose by stimulating hepatic glycogenolysis (Patel et al., 2013). Similarly, T3 treatment administered by intracerebroventricular injection (i.c.v.) increased endogenous glucose production via sympathetic stimulation of the liver in euthyroid rats (Klieverik et al., 2009) suggesting that the modulation of circulating glucose by T3 is multifaceted including direct neural stimulation. Additionally, in a diabetic condition, levels of glucose‐6‐phosphate (G6P) and 6‐PG dehydrogenase decreased compared to a non‐diabetic control, and treatment with thyroxine (T4) reversed these effects and increased hepatic G6P and 6‐glucose dehydrogenase activity (Glock & McLean, 1955). The rate of radioactive CO2 produced in hyperthyroid rats injected with C14‐6‐glucose was approximately double that of euthyroid controls suggesting that during a hyperthyroid condition, the rate of glucose oxidization is twice as high (Spiro & Ball, 1957). These data help reveal the diversity of TH actions on glucose metabolism via direct stimulation of hepatic glycogenolysis and of sympathetic activation of glucose production. Under insulin resistant conditions, hepatic G6P, which is the rate‐limiting step in glycolysis, is reduced; however, treatment with T4 rescued this effect. These data demonstrate the potential for THs to contribute to glucose metabolism via different pathways, helping to ameliorate some of glycolytic and oxidative impairments associated with insulin resistance in the liver.

#### 6.1.2 Skeletal muscle

Exogenous T4 improved glucose intolerance in obese, insulin resistant Otsuka Long Evans Tokushima Fatty (OLETF) rats that have otherwise normal functioning thyroid gland (Vazquez‐Anaya et al., 2017) suggesting that chronically (Tyagi et al., 2011) elevated T4 improves glucose intolerance and insulin resistance. It was proposed that this improvement in glucose metabolism was primarily mediated by improved insulin signaling in skeletal muscle. Similarly, a 10‐day treatment of T3 increased GLUT4 transcription by 70% in skeletal muscle (Torrance et al., 1997). Consistently, thyroidectomized rats showed reduced GLUT4 mRNA levels, which were restored by acute treatment with T3 in addition to increased GLUT4 trafficking to the plasma membrane (Brunetto et al., 2012). These data suggest that the improvements in glucose tolerance is, at least partially, attributed to improved GLUT4‐mediated glucose uptake in skeletal muscle.

#### 6.1.3 Brown adipose tissue

TH are key regulators in brown adipose tissue (BAT). In hyperthyroid patients, glucose uptake in BAT was 3‐fold higher compared to euthyroid condition (2.7 ± 2.3 vs. 0.9 ± 0.1 μmol/100 g/min) indicating BAT metabolism is modulated by TH (Lahesmaa et al., 2014). Other studies have revealed T3 stimulates mitochondrial activity in BAT. TH activates thermogenesis by inducing the mitochondrial uncoupling protein (UCP1). Expression of genes involved in thermogenesis such as UCP1 were significantly increased in hyperthyroid mice (Branco et al., 1999; Yau et al., 2019). Glucose is an essential contributor to BAT thermogenesis. In BAT, glucose is taken up by glucose transporters (GLUT1 & 4) and then undergoes glycolysis in the mitochondria where acetyl‐CoA undergoes partial breakdown into citrate. In the cytosol, citrate is then used in lipogenesis to generate TG from free FAs. Free FAs can activate UCP1 to generate heat (Hankir & Klingenspor, 2018). Consistently, a GLUT4‐inhibiting compound suppressed heat production in human brown adipocytes demonstrating the effects of glucose metabolism for BAT thermogenesis (Lee et al., 2016). Collectively, these data identify the effects of TH on glucose uptake in BAT and further increase thermogenesis. Furthermore, GLUT4 has a TRE in its promoter along with carbohydrate responsive element binding protein (ChREBP), retinoic acid receptors (RAR), and cAMP response element (CRE). Binding of T3 upregulated GLUT4, which translocates to the plasma membrane in brown adipocytes and sequesters glucose, ultimately regulating gluconeogenesis and glycolysis (Obregon, 2014). Additionally, T3 treatment enhanced GLUT4 content in adipose and soleus muscle (Panveloski‐Costa et al., 2020). Collectively, these data highlight the contributions of THs to the regulation of glucose metabolism in BAT and potentially thermogenesis and whole‐body energetics.

## 7 THS AND GLUCOSE INTOLERANCE

In Goto‐Kakizaki (GK) rats, a model of T2DM, intraperitoneal (i.p.) injections of T3 (1.5 μg/100 g of body mass), decreased serum TSH concentration by 75% compared to Wistar control rats. GK rats treated with T3 also had a reduced insulin response to a glucose challenge, with glucose decreasing 50% between 30 to 120 min of a glucose tolerance test (GTT) suggesting that insulin sensitivity improved in response to T3 treatment. Furthermore, patients with T2DM had a 10% prevalence of developing subclinical hypothyroidism when compared to healthy, euthyroid controls (Han et al., 2015) suggesting that impaired glucose metabolism is associated with abnormalities in TH levels. In a case study, a patient with T2DM, metabolic syndrome, and hypothyroidism was first treated with insulin followed by LT4 (Harbuwono et al., 2016). After six months of LT4 therapy, fasting blood glucose decreased by 54 mg/dL and by 165 mg/dL 2‐h post‐prandially suggesting that THs contribute to glucose metabolism and that replacement therapy may ameliorate the glucose intolerance associated with metabolic derangement. Nevertheless, TH treatment under insulin resistant conditions increase glucose uptake as seen by the decrease in plasma glucose levels and reduced insulin response indicating the potential for THs to improve insulin sensitivity and regulate glucose tolerance.

## 8 HUMAN AND ANIMAL STUDIES

The summary of data used in this review indicates that inconsistencies in the literature exist. It is important to note that animal studies primarily rodents do not necessarily reflect human conditions. The underlying factors which cause hyper‐ and hypothyroidism in humans are not always replicated accurately in animals. Therefore, the inconsistencies of TH effects on glucose metabolism when comparing humans to animals might reflect the inabilities of animal studies to properly represent the effects associated with these conditions with respect to studying glucose metabolism in the heart or other tissues during insulin resistance.

### 8.1 TH effects on myocardial glucose metabolism

The data on TH on glucose metabolism in the heart during T2DM conditions are sparse and this area of research warrants further examination. During starvation or diabetic conditions increased levels of pyruvate dehydrogenase kinase (PDHK), specifically PDHK4, phosphorylates and inactivates PDHK whose main function is to convert pyruvate to acetyl CoA to conserve glucose. Treatment with T3 increased cardiac PDHK activity in the mitochondria (Priestman et al., 1996) demonstrating the potential of TH to regulate substrate metabolism in the heart. Additionally, in a hyperthyroid condition pyruvate transport increased across the mitochondrial membrane (Paradies & Ruggiero,), while in a hypothyroid condition, pyruvate oxidation decreased suggesting that pyruvate transport/metabolism is sensitive to wide fluctuations in circulating TH (Paradies & Ruggiero, 1989). Furthermore, pyruvate transport across the mitochondrial membrane is dependent on membrane fluidity. Cardiolipin, an inner mitochondrial membrane phospholipid that contributes to membrane transport, was increased in hyperthyroid rats suggesting that TH can modulate mitochondrial pyruvate transport via membrane fluidity (Paradies & Ruggiero,). Additionally, decreased T3 impaired cardiac contractility through alterations in the gene expressions of sarcoplasmic and endoplasmic reticulum calcium ATPase 2 (SERCA2), which is a primary regulator of cardiac glucose transport (Waller et al., 2015). Conversely, supplementation with T3 reversed these effects (Katzeff et al., 1997). These findings highlight some direct effects of T3 on pyruvate transport, which is the last cytosolic step in glycolysis and allows pyruvate to enter the mitochondria where it is converted to acetyl CoA by PDHK. These findings reveal some of the mechanisms by which TH affect glycolysis and ATP production, and ultimately, cellular energetics. Additionally, decreased levels of TH reduce SERCA pumps leading to a decrease in glucose transport highlighting the effects of TH on cardiac function and providing evidence of the responsiveness of cardiomyocytes to TH. However, the effects of TH on cardiac function during insulin resistant and/or diabetic conditions are not defined.

Moreover, elevated T3 increased the rate of glucose oxidation and decreased the glycolytic intermediate, F6P, in cardiomyocytes (Burns & Reddy, 1975), while T3 treatment increased pyruvate oxidation in cardiac tissue (Bressler & Wittels, 1966). These findings highlight some of the inconsistencies of TH action on glycolysis as F6P is essential for the downstream conversion to pyruvate. A 1‐day treatment with T3 increased GLUT4 mRNA expression by 1.9‐fold and 3.8‐fold on day 6; however, these changes in gene expression did not translate into equivalent changes in GLUT4 protein expression. These findings suggest that treatment with TH increases the potential for GLUT4‐mediated transport of glucose and ultimately contribute to a shift in cardiac metabolism. However, because the mRNA levels did not reflect a parallel change in protein expression, it could not be determined if these changes in gene expression translated to changes in glucose trafficking across the plasma membrane. It is important to note that recent work has demonstrated chronic hyperthyroidism reduced the expression of PPARα, downregulating the generation of mitochondrial biogenesis and subsequently total energy production (Maity et al., 2013). Therefore, the effects of TH on substrate metabolism in the heart during diabetic conditions are inconsistent and not well‐defined, and as such, this area still remains largely unresolved.

Although studies of TH on glucose metabolism in the heart during diabetic conditions are sparse, understanding the contributions of THs on glucose metabolism in other peripheral tissues such as liver, skeletal muscle, and BAT may provide insights that can be extrapolated to the heart. Studies in other tissues have demonstrated a correlation between TH treatment and improved glucose metabolism suggesting that TH treatment has the potential to improve glucose metabolism in the heart during insulin resistance. Regardless, because GLUT4 has a TRE in its promoter region, TH should be able to regulate glucose metabolism in the heart via alterations in GLUT4. The few studies performed on cardiac tissue suggest that glucose metabolism, especially as it relates to pyruvate, is improved with T3 treatment. Contraction and systole is reliant on the energy produced by the TCA cycle and the hearts' ability to alternate between FA and glucose oxidation allows for efficient cardiac function. TH activity contributes to efficiency of cardiac contractility by activating the expression of myosin 6, and promoting mitochondrial biogenesis, mitochondrial protein synthesis, and increasing pyruvate transfer across mitochondrial membrane. These results in cardiac tissue along with previous studies on peripheral tissues suggest that TH have the potential to improve glucose metabolism by increasing glucose oxidation, glycogenolysis, and glucose uptake in cardiomyocytes during insulin resistance and the potential for TH to ameliorate diabetic cardiomyopathy. Nonetheless, the need for more studies around the potential of TH to improve glucose metabolism during impaired metabolic conditions remains.

Clinical and subclinical hyperthyroidism, as present in Graves' disease, is often accompanied by impaired glucose tolerance and insulin resistance, which is accompanied by increased metabolic rate and elevated glucose oxidation (Maratou et al., 2010). Thus, the effects of chronically elevated TH that characterize hyperthyroidism are not equivalent to increased TH induced by exogenous treatment suggesting that co‐existing perturbations associated with hyperthyroidism may mask the potential benefits of increased TH.

Nonetheless, further studies are needed to determine the specific mechanisms by which TH regulate glycolysis and glucose utilization in the heart during impaired metabolic conditions such as insulin resistance and diabetes. The incongruencies that appear in the literature contribute to a clear understanding of the effects of TH on glucose metabolism regardless of the tissue type. It is not clear what is happening in the heart during impaired metabolic conditions, therefore, to better understand the mechanisms that regulate substrate utilization during diabetic conditions in the heart, further research on the potential of TH to reconcile the impairment in glucose and FA metabolism associated with insulin resistance and T2DM is required.

## 9 GAPS IN KNOWLEDGE

Unfortunately, there remains much to be examined with respect to the effects of TH on substrate metabolism in the heart during conditions of metabolic disorders especially insulin resistance and T2DM. But given the pronounced shift to primarily lipid (FA) metabolism in the heart with the onset of diabetes, close examinations of TH on lipid and glucose metabolism are warranted with a particular focus on: (1) substrate regulating enzymes, (2) metabolite changes mapped by metabolomic approaches, and (3) direct analyses of mitochondrial function and biology. Correlating the changes in these variables to cardiac function would be especially important to understand the functional relevance of how the changes in substrate metabolism alters function and the potential for exogenous TH to ameliorate the impairments induced by metabolic defects.

## 10 CONCLUSION

The heart demands a constant supply of energy to maintain its continuous mechanical work and provide the systolic movement to maintain systemic and pulmonary blood flow. The heart derives its energy from substrates such as FA, glucose, and lactate. Under normal conditions cardiac tissue utilizes roughly 70% FA and 30% glucose for the production of ATP; however, during T2DM glucose metabolism is impaired and FA can account for up to 99% of energy production. Studies reveal that elevated plasma FA, TG, and myocyte lipid accumulation lead to the development of diabetic cardiomyopathy. Some major consequences of shifts in FA metabolism in the heart are increased oxidative stress and lipotoxicity, which may ultimately lead to mitochondrial dysfunction. Therefore, restoration of glucose metabolism during diabetic conditions is essential to maintain proper function and health of the heart. Recent studies on peripheral tissues such as skeletal muscle and adipose have demonstrated that TH treatment improved glucose intolerance in diabetic models and increased GLUT4 mRNA levels. GLUT4 has a TRE in its promoter suggesting that TH regulates GLUT4 gene expression. Elevated T3 increases glucose oxidation rate and decreases F6P in cardiomyocytes. Additionally, T3 increases pyruvate transport across the mitochondrial membrane and mitochondrial biogenesis. Collectively, studies on T3 in cardiac tissue suggest that TH may improve glucose metabolism during insulin resistance and ameliorate the detriments of diabetes and the metabolic syndrome. However, further studies are needed to determine the mechanisms by which TH's regulate glucose and lipid metabolism in the heart.

Acknowledgments

DAM was supported by USDA HSI Educational Grant 2017‐03691 during the writing of this review.

## Conflict of Interests

The authors declare no conflict of interests.

## AUTHOR CONTRIBUTION

DAM developed the topic idea, performed the literature searches, designed the review, generated the figures, and wrote the manuscript. RMO provided guidance on the topic, reviewed and edited drafts, and approved the final version of the manuscript.
